# The Evaluation of Dark Chocolate-Elicited Emotions and Their Relation with Physico Chemical Attributes of Chocolate

**DOI:** 10.3390/foods10030642

**Published:** 2021-03-18

**Authors:** Elena Bartkiene, Ernestas Mockus, Ema Monstaviciute, Jolita Klementaviciute, Erika Mozuriene, Vytaute Starkute, Paulina Zavistanaviciute, Egle Zokaityte, Darius Cernauskas, Dovile Klupsaite

**Affiliations:** 1 Institute of Animal Rearing Technologies, Lithuanian University of Health Sciences, Tilzes g. 18, LT-47181 Kaunas, Lithuania; elena.bartkiene@lsmuni.lt (E.B.); ernestas.mockus@lsmuni.lt (E.M.); jolita.klementaviciute@lsmuni.lt (J.K.); erika.mozuriene@lsmuni.lt (E.M.); vytaute.starkute@lsmuni.lt (V.S.); paulina.zavistanaviciute@lsmuni.lt (P.Z.); egle.zokaityte@lsmuni.lt (E.Z.); darius.cernauskas@lsmuni.lt (D.C.); 2 Department of Food Safety and Quality, Lithuanian University of Health Sciences, Tilzes g. 18, LT-47181 Kaunas, Lithuania; ema.monstaviciute@stud.lsmu.lt

**Keywords:** dark chocolate, emotions, overall acceptability, fatty acid profile, aromatic compounds

## Abstract

The aim of this study was to evaluate the effect of different origin (Venezuela, Ghana, Peru) dark chocolates on emotions induced for consumers, and to analyse the relationships among overall acceptability (OA), emotions, and physicochemical attributes of the chocolate (fatty acids (FAs) and volatile compounds (VC)). Chocolate-elicited emotions were measured with FaceReader 8 software, scaling 10 emotion patterns (neutral, happy, sad, angry, surprised, scared, disgusted, contempt, valence, arousal). The OA was carried out by using a 10-point hedonic scale, ranging from 1 (extremely dislike) to 10 (extremely like). The obtained results showed that, among all chocolate-elicited emotions, the intensity of “happy” was the highest. In most cases, the influence of the different origin chocolate on the emotions induced for consumers was significant (except on emotions “neutral”, “scared”, and “disgusted”). Significant differences between the tested chocolates OA were not found. The origin of chocolate had a significant effect on most of the identified VC and the content of the main FAs (methyl palmitate, methyl stearate, *cis,trans*-9- oleic acid methyl ester, and methyl linoleate). Significant correlations between chocolate-elicited emotions and separate FAs and VC were found. Finally, this study showed that the origin of dark chocolate significantly influenced most of chocolate-elicited emotions and physicochemical attributes of chocolate, while separate FAs or VC can be used as chocolate quality indicators related to the chocolate OA, as well as chocolate-elicited emotions.

## 1. Introduction

Consumers’ satisfaction with a food product is a very important characteristic that can lead to the product’s popularity, or in contrast, unpopularity, and stimulate consumers to return or, in contrast, never choose this product again in the future [1]. The sensory properties of chocolate make it one of the favourite treats worldwide. Recently, the consumption of dark chocolates increased, because of its health benefits described in literature [2,3,4]. It was reported that a high content of polyphenols in cocoa possesses desirable biological effects: antiatherosclerotic, anti-inflammatory, and regulation of the blood pressure and immune-response [5]. However, studies about the chocolate health benefits are controversial. It is important to point out that most chocolate is high in fat and free sugars, so should be consumed less often and in small amounts [6]. As well as dark chocolate is high in calories and can contribute to weight gain if eaten in excess [7]. It is difficult to be sure about the potential health effects and eating modest quantities may offer the greatest health benefits [8]. High-quality evidence for significant health benefits of dark chocolate has not been shown [9,10].

The most important sensory properties of the chocolate are associated with its texture and odour [2], which are related to the fatty acid (FA) and volatile compounds (VC) profile, respectively. Due to cacao butter polymorphism, chocolate tempering is a very important process, which helps to reach the wanted crystalline state of the cocoa butter, glossy look, well snap, contraction, and increases shelf life [11], and, the melting characteristics of the dark chocolate are influenced by the fat content, the FAs profile, particle size, emulsifiers used, and storage time, as well as conditions [11,12,13]. All of these parameters have an influence on the chocolate quality. In addition, characteristics of cacao mass differing in relation to cacao origin, which is depending on climate conditions, harvest time, and agricultural method applied [14]. Torres-Moreno et al. [15] observed that the FAs profile of dark chocolate produced from cocoa mass from Ghana and from Ecuador, is different, and this is associated more with geographical origin than the processing effect.

The odor of chocolate is also an essential quality property, which can be formed by more than 500 VC of different chemical classes [16,17]. The VC of the chocolate are influenced by the origin of the cocoa beans, technological treatment and the growing conditions [16,18,19]. However, it was reported that the fruity and floral notes of some esters and alcohols are stable during the dark chocolate preparation, and for this reason, the final product has the odour parameters typical for cacao bean [20].

Finally, both, texture and odor, are related with cocoa mass FA and VC profile, and affect the sensory characteristics, which can lead to different responses of the emotions induced for consumers [3]. The study of emotions in sensory and consumer research has grown rapidly in the past 10 years [21,22,23,24]. Food products can induce various emotional states and that depends on many aspects, including physiological, psychological, and sociological factors [25]. Food-elicited emotions might be also an essential factor when choosing a particular product because hedonic scale does not always thoroughly foresee food choices [26]. Diverse definitions of emotion are used by researchers and there is no single measurement that could cover all range of these aspects. Emotions can be measured using physiological techniques, facial analysis, verbal and visual test [27]. Various verbal self-reporting questioners have been commonly used in measuring emotions over the last two decades but the direct automatic measure of facial expressions in person face is still a less uncomfortable and a more natural method of emotions measurement [25,26]. Moreover, explicit methods, such as verbal or visual questionnaires, are fast but might be cognitively biased, while implicit methods, e.g., measurement of facial expressions, are indirect and lack the demand of a cognitive conversion [28]. However, the combination of implicit and explicit measurement techniques could provide comprehensive results but till now no standard combination is created [26]. Therefore, a better understanding of food induced emotions requires further studies in this field.

It was reported that the consumption of chocolate could diminish negative mood while “happy” and “surprise” emotions were frequently related with a sweet taste [29]. In our previous study, food-elicited emotions were measured using facial expression recognition software and results showed that the milk chocolate had the highest intensity of emotion “happy”, and reduced the level of the neutral state [30]. It was also concluded that Noldus FaceReader technology was sufficiently accurate to detect significant differences in facial expressions elicited by different samples of sugar confectionery products, such as chocolates, sweets, caramel [31]. As these food products evoke emotions, particular compounds in them could correlate with the elicited emotions. Therefore, in this study, we hypothesized, that the knowledge concerning the relation of the sensory/emotional responses with separate FA and VC profile compounds could bring additional value in food and emotion related studies, as well as to the food industry. Furthermore, a prognosis about the product popularity at the market could be made, and technological steps taken for chocolate quality improvement.

The aim of this study was to evaluate the effect of different origin (Venezuela, Ghana, Peru) dark chocolates on emotions induced for consumers, and to analyse the relationships among overall acceptability, emotions, and physicochemical attributes of the chocolate: fatty acids and volatile compounds.

## 2. Materials and Methods

### 2.1. Samples of the Dark Chocolate Preparation

**Figure 1:**
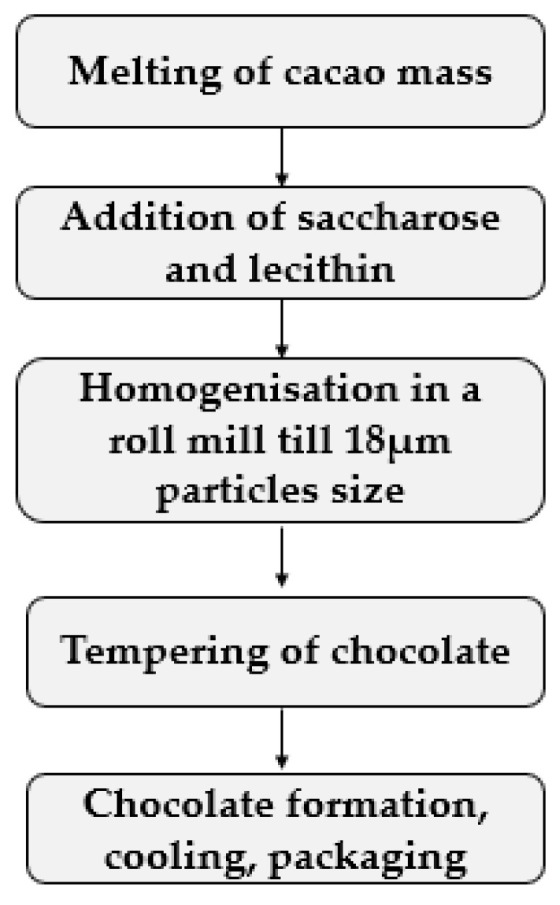
Technological scheme of chocolate preparation.

In total, 4 samples of the dark chocolate, by using different cocoa mass (country of origin Venezuela (KÖLLA Valencia S.L., Valencia, Spain)—samples group S1, country of origin Ghana (KÖLLA Valencia S.L., Valencia, Spain)—samples group S2, country of origin Peru: ecological—samples group S3 (Natra cocoa S.L., Valencia, Spain) and non-ecological—samples group S4 (KÖLLA Valencia S.L., Valencia, Spain)) were prepared at laboratory scale. Dry matter of cacao mass was 70%. Recipe of chocolate consist of 70% of cacao mass, 29.6% saccharose (obtained from “Nordic Sugar”, Kedainiai, Lithuania), 0.4% sunflower lecithin (obtained from “Naturz Organics”, Helmond, The Nederlands). The technological scheme of chocolate preparation is shown in Figure 1. Melting of the cacao mass was performed at 38–40 °C. Then, saccharose and lecithin were added, and a mass was homogenised in a ball mill (CAO B5: laboratory ball mill refiner, “Caotech b.v. Grinding Technology”, Wormerveer, The Nederlands). Chocolate mass was tempered, filled to the silicon molds, and cooled at 4 °C. Before the experiment, chocolate mass was kept in the dark place at 21 ± 2 °C.

### 2.2. Evaluation of the Dark Chocolate Overall Acceptability and Emotions Induced for Consumers

**Figure 2:**
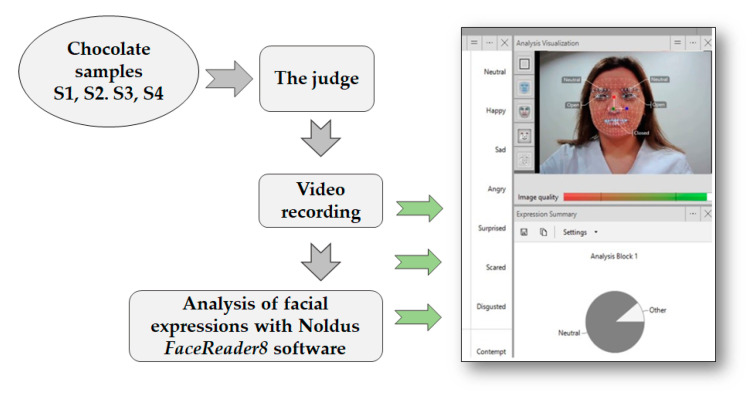
The experimental scheme used to evaluate the emotions elicited by different chocolate samples. Permission to use the original picture is provided by the coauthor E. Zokaityte.

The analysis was performed at the Lithuanian University of Health Sciences Sensory laboratory, which is equipped with sensory booths. Dark chocolate samples were evaluated by 30 panelists (age from 25 till 50 years, 15 women and 15 men). Mammasse and Schlich [32] reported that the suitable number of panelists could range from 20 to 150. depending on the level of complexity among test samples. Therefore, it should be noted that a small number of panelists might be a limitation of this study. Chocolate samples were prepared as pieces and presented in four separate serving plates with different codes. The panelist tasted the presented samples one by one in front of a webcam (Microsoft Corporation, Redmond, WA, USA). The tasting procedure was recorded. After tasting each sample, the panelist raised their hand and visualized the taste experience with a facial expression. The time for that was not limited. After that, the panelist was asked to evaluate the overall acceptability of sample using a 10-point hedonic scale, ranging from 1 (extremely dislike) to 10 (extremely like). Between samples, the panelists were asked to rinse the mouth with warm (40 ± 2 °C) water. To evaluate the chocolate-elicited emotions (neutral, happy, surprised, sad, scared, angry, contempt, arousal, disgusted, and valence), the recorded videos were analysed with FaceReader 8 software (Noldus Information Technology, Wageningen, The Netherlands). Only part of video when panelist raise his hand was used for the analysis of chocolate-elicited emotions. The intensity of each emotion was expressed in a scale from 0 (no emotion) to 1 (highest intensity of emotion). The experimental scheme used to evaluate the emotions elicited by different chocolate samples is given in Figure 2.

### 2.3. Fatty Acid Profile Analysis

The extraction of lipids for fatty acids (FA) analysis was done with chloroform/methanol (2:1 *v*/*v*) and fatty acid methyl esters (FAME) were prepared according to Pérez-Palacios et al. [33]. FA composition of the dark chocolate was identified using a gas chromatograph GC -2010 Plus (Shimadzu corp.) equipped with Mass Spectrometer GCMS-QP2010 (Shimadzu corp.). Separation was carried out on a Stabilwax-MS column (30 m length, 0.25 mmID, and 0.25 μm *df*) (Restek). Oven temperature programming started at 50 °C, it was raised 8 °C/min to 220 °C, held for 1 min at 220 °C, increased again at 20 °C/min to 240 °C, and held for the last 10 min. Injector temperature was 240 °C, interface −240 °C, and ion source 240 °C. The carrier gas was helium at a flow rate of 0.91 mL/min. The Individual FAME peaks were identified by comparing their retention times with those of standards (Merck & Co., Inc., Kenilworth, NJ, USA).

### 2.4. Analysis of Volatile Compounds by GC-MS Method

The volatile compounds (VC) in chocolate samples were analysed by gas chromatography-mass spectrometry (GC-MS) as described in Bartkiene et al. [34] with some modifications. Solid phase microextraction (SPME) device with Stableflex (TM) fiber coated with 85 µm PDMS-Carboxen™ layer (Supelco, Bellefonte, PA, USA) was used to prepare samples. For headspace extraction, 2 g of sample in the 20 mL extraction vial sealed with polytetrafluoroethylene septa was thermostated at 60 °C for 15 min, exposing the fiber in the headspace. The fiber was exposed to the headspace of the vial for 10 min. The desorption time was 2 min. Prepared samples were analysed with a GCMS-QP2010 (Shimadzu, Kyoto, Japan) gas chromatograph and a mass spectrometer. The following method conditions were used for analysis: injector temperature 250 °C, ion source temperature 220 °C, interface temperature 280 °C. Sample injection was carried out for 2 min on order to ensure full desorption of volatiles from the SPME fiber. The temperature gradient was programmed from start at 35 °C (5 min hold) to 200 °C (10 °C/min) up to 280 °C (25 °C/min) (5 min hold). The VCs were identified according to the mass spectra libraries (NIST11, NIST11S, FFNSC2).

### 2.5. Statistical Analysis

The results were expressed as the mean ± standard deviation (SD). The data were analyzed using the statistical package SPSS for Windows (v15.0, SPSS Inc., Chicago, IL, USA). The normal distribution of data was checked using Descriptive Statistics tests. In order to evaluate the influence of the different type of dark chocolate on induced emotions, FA profile, and aromatic compounds, data were analyzed by the one-way ANOVA and Tukey’s honestly significant difference (HSD) procedure, as post-hoc tests. A linear Pearson’s correlation was used to quantify the strength of the relationship between the variables (0.00–0.19, very weak; 0.20–0.39, weak; 0.40–0.59, moderate; 0.60–0.79, strong; 0.80–1.0, very strong) [35]. The results were recognized as statistically significant at *p* ≤ 0.05.

## 3. Results and Discussion

### 3.1. Overall Acceptability and Emotions Induced by the Dark Chocolate for Consumers

Results of the overall acceptability test and emotions induced by the dark chocolate for consumers are shown in Table 1. Significant differences of the tested chocolate samples overall acceptability were not found, and, on average, the overall acceptability of the samples was 8.2 points. The dark chocolate elicited the highest intensity of happiness (on average, 0.746. In most of the cases, the influence of the different chocolate mass on the emotions response for consumers was significant (*p* ≤ 0.05) (except on emotions “neutral“, “scared“ and “disgusted“). Consumers felt slightly higher intensity of “happy” when consuming chocolate sample S2, following by samples S1, S4, and S3. The highest emotion “sad” expression was fixed by testing S4 sample (0.015). The lowest “angry” emotion was expressed by testing S2 sample, however, by testing S1, S3, and S4 groups, emotion “angry” was fixed by 7, 12, and 6 times, respectively, more intensive. Consumers felt the lowest intensity of emotion “surprised” by testing S2 samples (0.004), also, S2 samples induced the lowest expression of the emotion “contempt”. Samples S3 induced, also, low expression of “contempt”, in comparing with S1 and S4 samples. Negative moderate correlation between the overall acceptability of the dark chocolate and emotion “contempt“ was established (*r* = 0.58) (Supplementary Table S1). Various sensory science methods have been developed to assess the emotions induced by food for consumers [36]. Most studies use explicit response measures by questionnaires that comprise a forced yes/no variant of CATA questions [37]. Food choice is related with a very complex function of preferences for sensory, as well as non-sensory attributes, including expectations and attitudes, price, ethical concerns, food—mood relation, and health claims [38,39,40]. One from the characteristics of chocolate is mood-enhancing properties, however, till now, opinion about which constituents may contribute to the psychopharmacological activities is controversial, and can be related with many compounds: flavanols, methylxanthines, salsolinol etc. [41]. Also, consumers tend to consume food as a form of self-expression, for lifestyle, image positioning etc. [42]. For this reason, the places where chocolate is sold also become very important and related with consumers’ choice [40].

However, in this case, only the emotion “contempt” correlated with overall acceptability, and this indicates that the facial expression analysis measures are not sensitive enough to distinguish between these emotional responses. Finally, further research is needed, in which more factors, related with the chocolate choice should be included. This is because such factors as craving [43,44] or feeling depressed [45] can lead to changes of the chocolate choice.

### 3.2. Fatty Acid Profile of the Dark Chocolate Samples

Fatty acid profile of the dark chocolate samples is shown in Table 2. The main FAs in all the tested chocolate samples were methyl palmitate (C16:0), methyl stearate (C18:0), *cis,trans*-9- oleic acid methyl ester (C18:0 *cis,trans*), and methyl linoleate (C18:2), and the different cacao mass, used for chocolate preparation, was significant factor on these FAs content (*p* ≤ 0.05). In comparing saturated (SFAs), monounsaturated (MUFAs), polyunsaturated (PUFAs), omega-3, 6, and 9 FAs in different chocolate samples, the highest content of SFAs in the S4 chocolate samples was found. The highest MUFAs, PUFAs, and omega-9 in S2 chocolate was established. On average, by 8.6, 1.9, and 5.0 times that in S1, S2, and S4 samples, respectively, the content of omega-3 in S3 samples was found, and the highest content of omega-6 in S1 samples was established.

Pearson correlations between the overall acceptability (OA) of the chocolate samples and separate FAs showed that there is a significant negative moderate correlation between OA and C16:0 (*r* = −0.62), and moderate positive correlations between OA and C18:0 (*r* = 0.66), C18:3ɣ (*r* = 0.69), C18:3α (*r* = 0.66), C20:0 (*r* = 0.80), and C20:1 (*r* = 0.78) (Supplementary Files Table S2). Also, significant correlations between the emotions induced for consumers by the tested chocolate samples and separate FAs were found. The highest number of significant correlations between the emotion “angry“ and separate FAs were found (between C8:0, C10:0, and C12:0 *r* = 0.89, between C14:0 *r* = 0.89, C16:1 *r* = 0.67, C17:0 *r* = 0.70, C18:0 *r* = −0.76, and C18:0 *cis*, *trans r* = −0.89), which means that emotion “angry” has a positive correlation with C8:0, C10:0, C12:0, C14:0, C16:1, and C17:0 fatty acids (by increasing the amount of these fatty acids in chocolate, the expression of “angry” increases, and, in opposite, by reducing above mentioned fatty acids in chocolate, expression of “angry” reduces), and, in opposite, negative correlation with C18:0 fatty acid means that increases of this fatty acid in chocolate, expression of “angry” reduces, or (and), by reducing C18:0 in chocolate, expression of “angry” increases. Between C16:0 and emotions “surprised“, “scared”, “disgusted“, and “contempt“ very strong positive (*r* = 0.83), moderate positive (*r* = 0.73), moderate negative (*r* = −0.66), and moderate positive (*r* = 0.70), respectively, correlations were found. Expressed emotions “scared”, “disgusted”, and “contempt” were correlated with FA C17:0 (*r* = 0.82, *r* = −0.46, and *r* = 0.75, respectively). Also, between the emotion “scared” and C18:0 *cis*, *trans*, C18: and C18:3ɣ FAs negative moderate correlations were found (*r* = −0.60, *r* = −0.71, and *r* = −0.70, respectively), as well as, in opposite, moderate positive correlations between the emotion “scared” and C24:0 was established (*r* = 0.64). Emotion “disgusted” showed positive correlations with C18:0 *cis*, *trans*, C18:3ɣ, C20:0 and C20:1 (*r* = 0.58, *r* = 0.73, *r* = 0.68, and *r* = 0.77, respectively). Between emotion “contempt” and C18:3ɣ negative strong correlation was found (*r* = 0.64), however, positive moderate and very strong correlations between the above-mentioned emotion and C22:0 and C24:0 FAs were established (*r* = 0.60 and *r* = 0.85, respectively).

In literature is reported that the main cocoa butter FA are stearic acid (34.62–35.67%), oleic acid (32.18–34.43%), and palmitic acid (25.24–28.41%) [46]. The differences of the cacao mass FAs profile in literature are mainly explained by the influence of geographical origins, but not by the chocolate processing conditions [15]. The main part of cocoa butter FA are TriAcylGlycerols, mainly comprising three key symmetrical TriAcylGlycerols (saturated–unsaturated-saturated) account for over 90% of all cocoa butter, including 1-Palmitate-2-Oleate-3-Stearate triacylglycerol, 1,3-diStearate-2-Oleate triacylglycerol, and 1,3-diPalmitate-2- Oleate triacylglycerol, with incidence depending on the cocoa’s geographic [14,47]. TriAcylGlycerols of cocoa are crystallized in different polymorphic forms that are differentiated at their melting point [13]. High quality of chocolate needs particular a polymorphic fat crystal that provide eligible physical properties [48,49]. Our study is the first, which analyzed the relations between separated FAs and sensory characteristics, as well as emotions induced for consumers during the chocolate testing. The obtained results clearly showed that separate FAs can be used as chocolate quality indicators related not just directly to texture parameters, but also to the emotions induced for consumers.

### 3.3. Volatile Compounds Profile of the Dark Chocolate

The volatile compounds (VC) profile (volatile acids, alcohols, aldehydes, esters, ketones, pyrazines, furans, among others) of the dark-chocolate samples was evaluated in order to identify possible key aroma markers associated with the different chocolate samples, prepared from the different cacao mass, as well as to evaluate which VC has the strongest correlations with overall acceptability and emotions induced for consumers (Supplementary Table S3). It was reported that several VC of the chocolate aroma are formed as a result of the technological processes [16,50]. However, the short-chain carboxylic acid, acetic acid, was the main VC identified in all the analysed chocolate samples, with the highest percentage in S4, and the lowest in S2 samples (78.23 and 67.90%, respectively). Acetic acid is the most intensive cacao VC profile compound, characterized by a vinegar-like odor [51]. The percentage of VC was varied in the different chocolate samples, and different cacao mass was a significant factor most of the identified VC (*p* ≤ 0.05). Out of the 119 VC identified, 29 were found in all, out of the four analysed, chocolate samples. Sample S3 showed the highest percentage of 2,3-butanediol, which odor is described as fruity, creamy, and buttery, however, 2,3-butanediol correlation with emotion “happy” was negative moderate (*r* = −0.636). As well as a negative correlations between the 2,3-butanediol and emotions “sad”, “angry”, “surprised”, “scared”, and “valence” were found (*r* = -0.721, *r* = -0.598, *r* = -0.859, *r* = -0.695, and *r* = -0.901, respectively). Also, 2,3-butanediol, [R-(R*, R*)] was found in all the tested chocolate samples, as well as a very strong positive correlation between this VC and “sad” emotion was established (*r* = 0.884).

In comparing the typical VC for separate chocolate samples in S1, S2, S3, and S4, out of all identified VC, there were 16, 15, 9, and 16, respectively, VC were typical just for particular sample. In S1 samples, despite a common VC identified in other samples, the main typical VC were 2,3-butanediol, [S-(R*, R*)], which odor is described as fruity and rum-like (2.60%); butanoic acid, 3-methyl-, whose odor is described as rancid and cheesy (0.41%); 2-heptanol (0.48%), which is associated with mushroom odor; sulfurous acid, isobuthyl 2-pentyl ester (0.62%), whose odor is described as orange juicy, impacting, musty green, unripe, fruity, reminiscent of banana and vegetative nuances with a slight nutty note; nonanal (0.40%), which odor is described as waxy, aldehydic, citrus, with a fresh slightly green lemon peel like nuance, and a cucumber fattiness, and other VC in lower percentages. Further, 2,3-Butanediol is eligible VC in high-quality cocoa products because it has a good stability during the production of chocolate [20,50].

In S2 samples, the main typical VC were acetic acid, ethoxyhydroxy- ethyl ester (1.12%), which odor is described as ethereal, fruity, sweet, weedy, and green; lactate (ethyl) (3.35%), which odor is described as sweet, fruity, acidic, etherial; tetradecane (n) (0.26%), which odor is described as mild waxy, and other VC in lower percentages. Samples S3 group was specific, because just in this group 1-butanol, 3-methyl-, acetate (0.13%), which odor is described as sweet, banana, fruity with a ripe estry nuance, and copaene (0.05%), which odor is described as woody, spicy, and honey, as well as other VC specific for this group in lower percentages, and (or), without odor characteristics. S4 group samples were the richest with the VC, which possessing specific odor: cyclohexanol, 4-(1,1-dimethyl)- (0.37%) with woody, musty, patchouli, camphor, mint, leather odor; pyrazine, trimethyl- (1.03%) with nutty, musty, powdery cocoa, potato and musty odor; octane, 2,3,3-trimethyl- (0.29%) with green, spicy, cilantro, fatty, leafy, cortex, herbal odor; nonane, 5-(2-methylpropyl)- (0.47%) with fruity, straw, caramel burnt odor; pyrazine, 3-ethyl-3,5-dimethyl- (0.31%) with peanut, nutty, caramel, coffee, musty, cocoa, pyrazine and roasted odor; 2-nonanone (0.48%) with fruity, sweet, waxy, soapy, cheese, green herbaceous, coconut like odor; 2-isopryl-5-methylhes-2-enal (0.21%) with herbal, lavender, woody, green, blueberry, tomato odor; acetic acid, 2-phenylethyl ester (0.20%) with sweet, honey, floral rosy, a slight yeasty honey note with a cocoa and balsamic nuance odor, and other.

Pearson correlations between OA of the chocolate samples and separate VC showed that there is a significant negative very strong correlation between OA and butanoic acid, 3-methyl- (*r* = -0.81) and very strong positive correlations between OA and cyclohexene, 3-(1-methylpropyl)- (*r* = 0.83) and pyrazine, tetramethyl- (*r* = 0.81) (Supplementary Files Table S4). Also, significant correlations between the emotions induced for consumers by the tested chocolate samples and separate VC were found. The significant correlations between the emotion “happy“ and separate VC were found: strong negative correlation with 2,3-butanediol (*r* = −0.71), 1-butanol, 3-methyl-, acetate (*r* = −0.68), 2-hexanol, 5-methyl- (*r* = −0.73), pyrazine, 2,3-dimethyl- (*r* = −0.75), decane, 2,3,4-trimethyl- (*r* = −0.74), 2-heptanol, acetate (*r* = −0.63), heptadecane (*r* = −0.67), 1-heptanol, 2,4-diethyl (*r* = −0.68), cyclopentasiloxane, decamethyl- (*r* = −0.66), decane, 2,6,7-trimethyl- (*r* = −0.64), phenol, 2-methyl-5-(1-methylethyl-) (*r* = −0.63), copaene (*r* = −0.72), hexadecane, 1-iodo- (*r* = −0.68), cyclononasiloxane, octadecamethyl- (*r* = −0.72); strong positive correlation with cyclotetrasiloxane, octamethyl- (*r* = 0.61). Other correlations are detailed in Supplementary Table S3.

Chocolate taste and aroma is directly connected to the contents of volatile aroma compounds [52]. Such VC as alcohols, sulfides, and ketones may have a significant impact on chocolate taste and odor [53]. It has been reported that the presence of acetic acid, 2-methylpropanoic acid, 3-methylbutanoic acid and hexanoic acid was observed in dark chocolate [54]. Flowery and candy notes of dark chocolate are induced by the high content of alcohol, such as 2-phenylethyl alcohol, while 3-methyl-butanal and phenylacetaldehydes are the main compounds associated with malty and honey-like odor [1]. In general, there is a little information in literature about the relation between the acceptance, or even emotional response, of dark chocolate by consumers and the factors affecting it. It has been reported that the most liked chocolate by panelists was related with sweet and bitter taste due to the by the presence of flavor compounds, such as 2,3-butanediol and 2-methyl-1-butanol. However, chocolate with a higher content of acids was evaluated by the panelists as the less liked sample [55].

## 4. Conclusions

Nowadays, food-elicited emotions become more and more important element in the assessment of food products acceptability and popularity in market. Chocolate is one of the favourite products worldwide and its sensory properties are highly related with the profile of fatty acids and volatile compounds. Therefore, different FA and VC profiles might lead to different emotional responses in consumers. This study showed that the origin of cacao mass had a significant effect on most of the identified VC and the content of the main FAs (methyl palmitate, methyl stearate, *cis,trans*-9- oleic acid methyl ester and methyl linoleate) in tested chocolates. In most of the cases, the influence of the different origin chocolates on the emotions response for consumers was significant (except on “neutral”, “scared”, and “disgusted” emotions), although significant differences between different origin chocolates overall acceptability were not found. Also, significant correlations between the emotions induced for consumers by the tested chocolates and separate FAs and VC were found. In general, the intensity of “happy” emotion was the highest compared to other emotions. However, only the emotion “contempt” correlated with overall acceptability, and this indicates that the facial expression analysis measures are not sensitive enough to distinguish between these emotional responses. In this case, further research is needed, whereby more factors related with the chocolate choice and chocolate-induced emotions should be included. Finally, the obtained results of the relation of the sensory/emotional responses with separate chocolate FA and VC profile compounds can provide additional value in food and emotions related studies as well as in the food industry. Furthermore, a prognosis concerning product popularity on the market could be made, and technological steps for chocolate quality improvement can be taken.

## Figures and Tables

**Table 1:** Overall acceptability and emotion response of the dark chocolate samples.

Emotions Induced by the Chocolate (from 0 to 1)	Chocolate Samples			
	S1	S2	S3	S4
Overall acceptability	7.9 ± 0.8 ^a^	8.6 ± 0.7 ^a^	8.4 ± 0.9 ^a^	7.9 ± 0.7 ^a^
Neutral	0.002 ± 0.0003 a	0.001 ± 0.0002 ^a^	0.001 ± 0.0002 ^a^	0.001 ± 0.0002 ^a^
Happy	0.758 ± 0.019 ^ab^	0.763 ± 0.019 ^b^	0.721 ± 0.018 ^a^	0.742 ± 0.019 ^ab^
Sad	0.005 ± 0.0002 ^a^	0.009 ± 0.0003 ^b^	0.009 ± 0.0003 ^b^	0.015 ± 0.0005 ^c^
Angry	0.014 ± 0.001 ^b^	0.002 ± 0.0007 ^a^	0.024 ± 0.003 ^c^	0.012 ± 0.001 ^b^
Surprised	0.006 ± 0.0007 ^b^	0.004 ± 0.0008 ^a^	0.010 ± 0.004 ^c^	0.009 ± 0.0006 ^c^
Scared	0.001 ± 0.0008 ^a^	0.002 ± 0.0006 ^a^	0.001 ± 0.00003 ^a^	0.001 ± 0.0003 ^a^
Disgusted	0.001 ± 0.0003 ^a^	0.001 ± 0.0008 ^a^	0.001 ± 0.0006 ^a^	0.001 ± 0.0007 ^a^
Contempt	0.011 ± 0.0005 ^c^	0.006 ± 0.0007 ^a^	0.005 ± 0.0006 ^a^	0.008 ± 0.0009 ^b^
Valence	0.017 ± 0.0009 ^b^	0.011 ± 0.0008 ^a^	0.032 ± 0.002 ^d^	0.026 ± 0.002 ^c^

Data expressed as a mean value (*n* = 30) ± standard deviation. ^a–c^ Means with different letters within a row are significantly different (*p* ≤ 0.05).

**Table 2:** Fatty acid profile of the dark chocolate.

Fatty Acids	Chocolate Samples			
	S1	S2	S3	S4
	Fatty Acids Concentration, % from Total Fat Content			
C4:0	nd	nd	nd	nd
C6:0	nd	nd	nd	nd
C8:0	nd	nd	nd	0.336 ± 0.028
C10:0	nd	nd	nd	0.278 ± 0.054
C11:0	nd	nd	nd	nd
C12:0	nd	nd	nd	3.309 ± 0.986
C13:0	nd	nd	nd	nd
C14:0	0.051 ± 0.007 ^a^	0.068 ± 0.004 ^c^	0.057 ± 0.003 ^b^	1.419 ± 0.096 ^d^
C14:1	nd	nd	nd	nd
C15:0	nd	nd	nd	nd
C15:1	nd	nd	nd	nd
C16:0	25.868 ± 0.101 ^b^	23.211 ± 0.028 ^a^	25.948 ± 0.114 ^b^	25.738 ± 0.191 ^b^
C16:1	0.100 ± 0.009 ^a^	0.132 ± 0.012 ^b^	0.143 ± 0.011 ^b^	0.148 ± 0.018 ^b^
C17:0	0.144 ± 0.012 ^a^	0.130 ± 0.018 ^a^	0.176 ± 0.010 ^b^	0.207 ± 0.014 ^c^
C17:1	nd	nd	nd	nd
C18:0	32.553 ± 0.156 ^b^	33.659 ± 0.418 ^c^	33.022 ± 0.318 ^c^	29.581 ± 0.128 ^a^
C18:0 cis. trans	32.850 ± 0.110 ^c^	32.973 ± 0.132 ^c^	32.401 ± 0.118 ^b^	30.965 ± 0.098 ^a^
C18:2	7.153 ± 0.078 ^d^	6.917 ± 0.097 c	4.121 ± 0.234 ^a^	6.429 ± 0.118 ^b^
C18:2 trans	nd	nd	nd	nd
C18:3ɣ	nd	0.06 ± 0.01	nd	nd
C18:3α	0.379 ± 0.118 ^a^	1.730 ± 0.118 ^c^	3.256 ± 0.769 ^d^	0.647 ± 0.237 ^b^
C20:0	0.618 ± 0.008 ^a^	0.832 ± 0.019 ^c^	0.654 ± 0.004 ^c^	0.639 ± 0.009 ^b^
C20:1	0.053 ± 0.011 ^a^	0.125 ± 0.014 ^c^	0.055 ± 0.031 ^a^	0.058 ± 0.024 ^a^
C20:2	nd	nd	nd	nd
C20:3	nd	nd	nd	nd
C21:0	nd	nd	nd	nd
C20:4	nd	nd	nd	nd
C20:3	nd	nd	nd	nd
C20:5	nd	nd	nd	nd
C22:0	0.158 ± 0.003 ^d^	0.110 ± 0.005 ^b^	0.094 ± 0.008 ^a^	0.148 ± 0.005 ^c^
C22:1	nd	nd	nd	nd
C22:2	nd	nd	nd	nd
C23:0	nd	nd	nd	nd
C24:0	0.073 ± 0.006 ^b^	0.053 ± 0.011 ^a^	0.073 ± 0.008 ^b^	0.098 ± 0.010 ^c^
C22:6	nd	nd	nd	nd
C24:1	nd	nd	nd	nd
SFAs	59.465	58.063	60.024	61.753
MUFAs	33.003	33.23	32.599	31.171
PUFAs	7.532	8.707	7.377	7.076
Omega3	0.379	1.73	3.256	0.647
Omega6	7.153	6.977	4.121	6.429
Omega 9	33.003	33.23	32.599	31.171

C4:0—methyl butyrate; C6:0—methyl hexonoate; C8:0—methyl octanoate; C10:0—methyl decanoate; C11:0—methyl undecanoate; C12:0—methyl laurate; C13:0—methyl tridecanoate; C14:0—methyl tetradecanoate; C14:1—methyl myristoleate; C15:0—methyl pentadecanoate; C15:1—cis-10-pentadecenoic acid methyl ester; C16:0—methyl palmitate; C16:1—methyl palmitoleate; C17:0—methyl heptadecanoate; C17:1—cis-10-heptadecanoic acid methyl ester; C18:0—methyl stearate; C18:0 cis. trans—cis.trans-9- oleic acid methyl ester; C18:2—methyl linoleate; C18:2 trans–linolelaidic acid methyl ester; C18:3ɣ—gamma- linolenic acid methyl ester; C18:3α—alfa linolenic acid methyl ester; C20:0—eicosanoic acid methyl ester; C20:1—cis-11-eicosenoic acid methyl ester; C20:2—cis-11.14-eicosadienoic acid methyl ester; C20:3—cis-8.11.14-eicosatrienoic acid methyl ester; C21:0—methyl heinecosonoate; C20:4—cis-5.8.11.14-eicosatetraenoic acid methyl ester; C20:3—cis-11.14.17-eicosatrienoic acid methyl ester; C20:5—cis-5.8.11.14.17-eicosapentanoic acid methyl ester; C22:0—methyl docosanoate; C22:1—cis-13-docosenoic acid methyl ester; C22:2—cis-13.16-docosadienoic acid methyl ester; C23:0—methyl tricosanoate; C24:0—methyl tetracosanoate; C22:6—all cis-4.7.10.13.16.19-docosahexanoic acid methyl ester; C24:1—cis-15-tetracosenoic acid methyl ester; SFAs—saturated fatty acids ; MUFAs—monounsaturated fatty acids; PUFAs—polyunsaturated fatty acids. Data expressed as a mean value (*n* = 3) ± standard deviation. ^a–d^—Means with different letters within a row are significantly different (*p* ≤ 0.05).

## Supplementary Materials

The following are available online at https://www.mdpi.com/2304-8158/10/3/642/s1, **Table S1**: Pearson correlations between overall acceptability and emotion response of the dark chocolate samples; Table S2: Pearson correlations between overall acceptability and emotions response of the dark chocolate samples with fatty acids; Table S3: Volatile compounds (%) of the dark chocolate identified by HS-SPME GC-MS; Table S4: Pearson correlations between overall acceptability and emotions response of the dark chocolate samples with volatile compounds.
