# Supplementary material for: The Evaluation of Dark Chocolate-Elicited Emotions and Their Relation with Physico Chemical Attributes of Chocolate
Source: Foods. 2021 Mar 18;10(3):642. doi: 10.3390/foods10030642 (PMC8002928; doi:10.3390/foods10030642)
Supplement: Supplementary file 1 [file foods-10-00642-s001.pdf]

foods MDPI

Table S1. Pearson correlations between overall acceptability and emotion response of the dark chocolate samples.

		Overall acceptability
**Overall Acceptability**	Pearson Correlation	1
	Sig. (2-tailed)	
**Neutral**	Pearson Correlation	-0,265
	Sig. (2-tailed)	0,405
**Happy**	Pearson Correlation	0,310
	Sig. (2-tailed)	0,327
**Sad**	Pearson Correlation	-0,083
	Sig. (2-tailed)	0,796
**Angry**	Pearson Correlation	-0,126
	Sig. (2-tailed)	0,695
**Surprised**	Pearson Correlation	-0,091
	Sig. (2-tailed)	0,778
**Scared**	Pearson Correlation	0,496
	Sig. (2-tailed)	0,101
**Disgusted**	Pearson Correlation	-0,167
	Sig. (2-tailed)	0,603
**Contempt**	Pearson Correlation	-0,580*
	Sig. (2-tailed)	0,048
**Valence**	Pearson Correlation	-0,114
	Sig. (2-tailed)	0,723

*. Correlation is significant at the 0.05 level (2-tailed).

Table S2. Pearson correlations between overall acceptability and emotions response of the dark chocolate samples with fatty acids.

Fatty acids	Overall acceptability		Happy		Sad		Angry		Surprised		Scared		Disgusted		Contempt		Valence	
	r	*p*	*r*	*p*	*r*	*p*	*r*	*p*	*r*	*p*	*r*	*p*	*r*	*p*	*r*	*p*	*r*	*p*
**C8:0**	-0.492	0.104	-0.306	0.334	-0.102	0.752	0.887**	0.0001	-0.073	0.822	0.393	0.206	-0.414	0.181	0.235	0.462	0.123	0.704
**C10:0**	-0.491	0.105	-0.305	0.335	-0.101	0.756	0.887**	0.0001	-0.072	0.823	0.394	0.205	-0.413	0.182	0.237	0.458	0.123	0.702
**C12:0**	-0.492	0.104	-0.306	0.334	-0.103	0.751	0.887**	0.0001	-0.073	0.822	0.393	0.206	-0.414	0.18	0.234	0.464	0.122	0.705
**C14:0**	-0.484	0.11	-0.312	0.323	-0.1	0.758	0.890**	0.0001	-0.079	0.806	0.39	0.21	-0.41	0.186	0.228	0.475	0.116	0.719
**C16:0**	-0.624*	0.03	0.314	0.32	-0.452	0.14	0.035	0.914	0.832**	0.001	0.733**	0.007	-0.661*	0.019	0.700*	0.011	0.345	0.273
**C16:1**	0.502	0.096	-0.376	0.228	0.124	0.701	0.666*	0.018	0.168	0.602	0.551	0.063	-0.054	0.867	0.34	0.28	-0.4	0.198
**C17:0**	-0.116	0.719	-0.093	0.774	-0.051	0.875	0.700*	0.011	0.467	0.126	0.824**	0.001	-0.455	0.137	0.748**	0.005	0.039	0.904
**C18:0**	0.657*	0.02	0.126	0.697	0.148	0.647	-0.763**	0.004	-0.079	0.807	-0.462	0.131	0.499	0.098	-0.367	0.241	-0.288	0.364
**C18:0 *cis. trans***	0.478	0.116	0.377	0.227	0.307	0.332	-0.891**	0.0001	-0.171	0.595	-0.595*	0.041	0.577*	0.05	-0.33	0.295	0.005	0.989
**C18:2**	-0.277	0.383	0.447	0.145	0.703*	0.011	-0.135	0.675	-0.784**	0.003	-0.709**	0.01	0.508	0.092	-0.246	0.44	0.670*	0.017
**C18:3γ**	0.688*	0.013	-0.275	0.386	0.486	0.109	-0.074	0.818	-0.785**	0.002	-0.702*	0.011	0.730**	0.007	-0.683*	0.014	-0.344	0.274
**C18:3α**	0.660*	0.02	-0.519	0.084	-0.479	0.115	-0.038	0.907	0.463	0.129	0.35	0.264	-0.147	0.648	-0.069	0.832	-0.846**	0.001
**C20:0**	0.797**	0.002	-0.343	0.275	0.467	0.125	0.007	0.983	-0.689*	0.013	-0.563	0.057	0.680*	0.015	-0.571	0.053	-0.442	0.151
**C20:1**	0.779**	0.003	-0.179	0.578	0.647*	0.023	0.01	0.976	-0.696*	0.012	-0.542	0.069	0.769**	0.003	-0.419	0.175	-0.264	0.407
**C22:0**	-0.361	0.249	0.718**	0.009	0.660*	0.02	0.042	0.897	-0.065	0.84	0.088	0.785	0.105	0.745	0.597*	0.04	0.857**	0.0001

r – Pearson correlation; p – significance; correlation significant. when p≤0.05 (*) or p≤0.01 (**).

Table S3. Volatile compounds (%) of the dark chocolate identified by HS-SPME GC-MS.

Volatile compounds	Odour description	Retention time, min	Dark chocolate samples group			
			S1	S2	S3	S4
			Volatile compound peak area, %			
Acetic acid	Sour, vinegary	3.128	71.61 ±1. 90a	67.90 ±2. 16a	70.53 ±2. 34a	78.23 ±2. 41b
Propanoic acid, 2-methyl-	Fruity, rum-like	6.605	nd	1.82 ±0. 29b	nd	0.14 ±0. 03a
2,3-Butanediol, [R-(R*, R*)]	Fruity, creamy, buttery	6.694	2.17 ±0. 20a	2.14 ±0. 18a	1.84 ±0. 19a	2.62 ±0. 11b
2,3-Butanediol	Fruity, creamy, buttery	6.707	nd	nd	6.17 ±0. 89b	1.49 ±0. 29a
Acetic acid, ethoxyhydroxy-ethyl ester	Ethereal fruity sweet weedy green	6.860	nd	1.12±0. 29	nd	nd
Lactate <ethyl>	Sweet, fruity, acidic, etherial	6.920	nd	3.35 ±0. 92	nd	nd
2,3-Butanediol, [S-(R*, R*)]	Fruity, rum-like	6.950	2.60 ±0. 78	nd	nd	nd
Threo-3-bromo-2-pentanol	-	7.560	nd	nd	nd	0.19 ±0. 03
Butanoic acid, 3-methyl-	Sweet, diffusive, estry, fruity, sharp, pineapple, apple, green and orange	8.819	1.70 ±0. 25c	nd	0.06 ±0. 01a	1.02 ±0. 29b
Pentanoic acid, 3-methyl-	Characteristic, fresh notes of walnut, dry, woody	9.004	nd	0.05 ±0. 01	nd	nd
Butanoic acid, 3-methyl-	Rancid, cheesy	9.005	0.41 ±0. 10	nd	nd	nd
Butyric acid <2-methyl->	Acidic, fruity, dirty, cheesey with a fermented nuance	9.079	0.44 ±0. 11b	0.43 ±0. 10b	0.43 ±0. 10b	0.19 ±0. 05a
1-Butanol, 3-methyl-, acetate	Sweet, banana, fruity with a ripe estry nuance	9.357	nd	nd	0.13 ±0. 04	nd
L-lactic acid	Odorless	9.490	0.05 ±0. 01a	1.29 ±0. 21b	nd	nd
Pentanoic acid	Sweat	9.655	0.39 ±0. 09c	0.27 ±0. 04b	0.31 ±0. 05b	0.14 ±0. 05a
2-Butenoic acid, 3-methyl-	Green phenolic dairy	9.765	0.12 ±0.03	nd	nd	nd
2-Heptanol	Mushroom	9.860	0.48 ±0. 08b	nd	nd	nd
2-Hexanol, 5-methyl-	-	9.892	nd	nd	0.55 ±0. 11b	0.21 ±0. 08a
Pyrazine, 2,3-dimethyl-	Musty, nut skins, cocoa powdery and roasted with potato and coffee nuances	10.295	0.30 ±0. 07b	0.15 ±0. 08a	0.55 ±0. 10c	0.46 ±0. 06c
1-Methoxy-2-propyl acetate	Mild odour	10.537	0.40 ±0. 13a	1.44 ±0. 17b	0.64 ±0. 12a	0.59 ±0. 11a
Pentanoic acid, 4-methyl-	Pungent, cheese-like	11.066	0.07 ±0. 03b	0.02 ±0. 01a	0.12 ±0. 04b	0.09 ±0. 02b
Benzaldehyde	Strong sharp sweet bitter almond cherry	11.250	0.07 ±0. 01a	0.73 ±0. 10c	0.92 ±0. 11c	0.60 ±0. 10b

Hexanoic acid	Cheesy fruity phenolic fatty goaty	11.788	0.86 ±0. 08b	0.68 ±0. 08a	0.65 ±0. 10a	0.51 ±0. 12a
Heptane, 2,2,4,6,6-pentamethyl-	-	11.909	0.49 ±0. 09	nd	nd	nd
Cyclohexanol, 4-(1,1-dimethyl)-	Woody musty patchouli camphor mint leather	11.916	nd	nd	nd	0.37 ±0. 11
Cyclohexene, 3-(1-methylpropyl)-	Raw green herbal	11.920	nd	0.40 ±0. 10a	0.41 ±0. 11a	nd
Cyclotetrasiloxane, octamethyl-	-	12.056	0.42 ±0. 08a	0.40 ±0. 07a	nd	nd
Pyrazine, trimethyl-	Nutty, musty, powdery cocoa, potato and musty	12.136	nd	nd	nd	1.03 ±0. 11b
(+)-4-Carene	-	12.337	0.10 ±0. 02	nd	nd	nd
Octane, 2,3,3-trimethyl-	Green, spicy, cilantro, fatty, leafy, cortex, herbal	12.354	nd	nd	nd	0.29 ±0. 05
Heptane, 2,5,5-trimethyl-	-	12.540	nd	0.07 ±0. 01	nd	nd
Decane, 2,3,4-trimethyl-	-	12.547	nd	nd	0.08 ±0. 03	0.04 ±0. 02a
2-Heptanol, acetate	Fresh lemon grass, herbal, sweet, floral, fruity, green	12.905	0.01 ±0. 001a	nd	0.06 ±0. 01b	0.07 ±0. 01b
Pentane, 2,2-oxybis-	Sweet	13.140	nd	0.11 ±0. 01	nd	nd
Pentadecane	Waxy	13.225	nd	0.26 ±0. 03	nd	nd
Heptadecane	-	13.230	nd	nd	0.32 ±0. 06	nd
Nonane, 5-(2-methylpropyl)-	Fruity, straw, caramel burnt	13.237	nd	nd	nd	0.47 ±0. 07
Ethanone, 1-(1H-pyrrol-2-yl)-	Musty, nutty-like with a coumarin nuance	13.293	0.39 ±0.07a	0.31 ±0. 09a	0.36 ±0.08a	0.33 ±0. 06a
2-Pyrrolidinone	-	13.405	nd	nd	nd	0.08
Acetophenone	Sweet, cherry pit, marzipan and coumarinic, It has a slight almond nutty and heliotropin-like vanilla nuance	13.486	0.39 ±0.10a	0.34±0.08a	0.33 ±0.09a	nd
Heptanoic acid	Cheesy, waxy, sweaty, fermented, pineapple and fruity	13.531	nd	0.33±0.08b	0.34 ±0.07b	0.18 ±0.06a
Pyrazine, 3-ethyl-3,5-dimethyl-	Peanut, nutty, caramel, coffee, musty, cocoa, pyrazine and roasted	13.647	nd	nd	nd	0.31 ±0.07a
Pyrazine, 3-ethyl-2,5-dimethyl-	Potato, cocoa, roasted, nutty	13.652	0.23 ±0.07a	nd	0.37 ±0.09a	nd
H-Methyldodecanol	-	13.710	nd	0.07±0.02	nd	nd
1-Heptanol, 2,4-diethyl	-	13.720	nd	nd	0.15 ±0.03	nd
2-Undecene, 2,5-dimethyl	-	13.731	nd	nd	nd	0.09 ±0.02

Pyrazine, tetramethyl-	Nutty, musty and vanilla with dry, brown cocoa nuances	13.787	0.68 ±0.10a	1.57 ±0.17b	1.76 ±0.12b	0.79 ±0.11a
2-Nonanone	Fruity, sweet, waxy, soapy, cheese, green herbaceous, coconut like	13.840	nd	nd	nd	0.48±0.11
Sulfurous acid, isobuthyl 2-pentyl ester	Orange juicy, impacting, musty green unripe fruity reminiscent of banana and vegetative nuances with a slight nutty note	14.017	0.62 ±0.12	nd	nd	nd
1,6-Octadien-3-ol, 3,7-dimethyl-	Citrus, orange, floral, terpy, waxy and rose	14.019	nd	0.99 ±0.11a	0.94 ±0.12a	0.81 ±0.09a
Nonanal	Waxy, aldehydic, citrus, with a fresh slightly green lemon peel like nuance, and a cucumber fattiness	14.077	0.40 ±0.07	nd	nd	nd
2-Isopryl-5-methylhes-2-enal	Herbal, lavender, woody, green, blueberry, tomato	14.166	nd	nd	nd	0.21 ±0.08
Phenethyl alcohol	Sweet, floral, fresh and bready with a rosey honey nuance	14.299	0.50 ±0.07a	0.49 ±0.09a	0.61 ±0.09a	0.43 ±0.08a
1-Heptanol, 6-methyl-	-	14.606	0.05 ±0.01	nd	nd	nd
4H-Pyran-4-one, 2,3-dihydro-3,5-dihydroxy-6	-	14.804	nd	0.26±0.04	nd	nd
Cyclopentasiloxane, decamethyl-	-	14.896	0.54 ±0.09b	0.26 ±0.08a	1.99 ±0.17c	0.44 ±0.07b
Octanoic acid	Fatty, waxy, rancid, oily, vegetable, cheesy	15.224	0.69 ±0.10a	0.67 ±0.09a	0.72 ±0.10a	0.52 ±0.09a
3-Cyclohexen-1-ol, 4-methyl-1-(1-methylethyl)	Musty dusty	15.455	0.04 ±0.01a	nd	nd	0.02 ±0.01a
Octanoic acid, ethyl ester	Waxy, sweet, musty, pineapple and fruity with a creamy, dairy nuance	15.640	0.07 ±0.01a	nd	0.08 ±0.02a	0.07 ±0.01a
Tetradecane <n>	Mild waxy	15.708	nd	0.26±0.08	nd	nd
Dodecane	-	15.719	0.23 ±0.09	nd	0.26 ±0.07a	nd
Pentadecane <n>	Waxy	15.723	nd	nd	nd	0.14 ±0.03a
Estragole	Sweet, phenolic, anise, harsh, spice, green, herbal, minty	15.773	0.59 ±0.09b	0.57 ±0.08b	0.54 ±0.07b	0.33 ±0.06a
Sulfurous acid, decyl 2-ethylhexyl ester	-	15.938	nd	0.07±0.01	nd	nd
Undecane, 2,6-dimethyl-	-	15.945	0.05 ±0.01	nd	nd	nd
Decane, 2,6,7-trimethyl-	-	15.951	nd	nd	0.07 ±0.01a	0.05 ±0.01a
Thymoquinone	Pencil-like	16.613	0.35±0.08a	0.34 ±0.06a	0.35 ±0.06a	0.20 ±0.09a

Phenethyl isobutyrate	Heavy fruity, honey and yeasty, with balsamic nuances and waxy rosy floral notes on dry out	16.674	0.15 ±0.07a	0.19 ±0.04a	0.22 ±0.06a	nd
Acetic acid, 2-phenylethyl ester	Sweet, honey, floral rosy, with a slight yeasty honey note with a cocoa and balsamic nuance	16.678	nd	nd	nd	0.20 ±0.05
Nonanoic acid	Waxy, dirty and cheesy with a cultured dairy nuance	16.779	1.47 ±0.09b	1.59 ±0.09b	1.64 ±0.10b	1.28 ±0.07a
2H-Pyran-2-one, 5,6-dihydro-6-pentyl-	-	16.869	0.15 ±0.04a	0.10 ±0.03a	0.17 ±0.07a	0.09 ±0.03a
Hentriacontane <n->	-	16.993	0.09 ±0.02	nd	nd	nd
Dodecane, 4,6-dimethyl-	-	16.998	nd	0.10 ±0.04a	0.12 ±0.03a	0.17 ±0.04a
Phenol, 2-methyl-5-(1-methylethyl-)	Spicy, cooling, thymol-like, herbal and camphoreous with smoky nuances	17.195	1.02 ±0.12b	1.04 ±0.11b	1.03 ±0.12b	0.67 ±0.10a
Nonane, 5-methyl-5-propyl-	-		nd	nd	nd	0.03±0.01
Nonane, 3-methyl-5-propyl-	-	17.273	0.09 ±0.02b	nd	nd	0.04 ±0.01a
Pentadecane <n>	Waxy	17.279	nd	nd	0.09 ±0.02	nd
Tridecane <n>	-	17.283	nd	0.07±0.02	nd	nd
Thymol	Spicy, phenolic and thymol with a chemical, medicinal, camphoreous nuance	17.359	0.08 ±0.02a	0.09 ±0.02a	0.07 ±0.01a	0.07 ±0.01a
Octadecane, 1-(ethenyloxy-)	-	17.408	nd	nd	nd	0.06±0.01
Cyclohexasiloxane, dodecamethyl-	Odorless	17.614	0.52 ±0.11b	0.14 ±0.04a	0.23 ±0.06a	0.23 ±0.06a
Nonane, 3-methyl-5-propyl-	-	17.680	0.05 ±0.01a	nd	0.07 ±0.01a	nd
Eicosane	Waxy	17.689	nd	0.06±0.07a	nd	nd
Dodecane, 4,6-dimethyl-	-	17.691	nd	nd	nd	0.08±0.01
1-Alanine, N-cyclopropylcarbonyl-, methyl ester	-	17.863	0.02 ±0.01a	nd	0.03 ±0.01a	nd
3H-Pyrazol-3-one, 2,4-dihydro-2,5-dimethyl	-	17.871	nd	nd	nd	0.03±0.01
Sarcosine, N-cyclopropylcarbonyl-, octyl ester	Sweet, fruity, oily-waxy, with a green creamy nuance	17.867	nd	0.02±0.01	nd	nd
Decanoic acid <n->	Unpleasant rancid sour fatty citrus	18.195	0.30 ±0.06b	0.23 ±0.06ab	0.24 ±0.07ab	0.17 ±0.03a
Propanoic acid, 2-methyl-, 3-hydroxy-2,4,4-tri	-	18.487	0.44 ±0.07b	0.29 ±0.06a	0.23 ±0.06a	0.12 ±0.07a
Copaene	Woody, spicy, honey	18.626	nd	nd	0.05 ±0.01	nd
Hexadecane, 1-iodo-	-	18.734	nd	nd	0.14 ±0.02	nd
Methylengenol	-	18.845	0.08 ±0.02a	0.07 ±0.02a	0.06 ±0.02a	0.05 ±0.02a

2,4,7,9-Tetramethyl-5-decyn-4,7-diol	-	18.983	0.19 ±0.03b	0.17 ±0.04b	0.18 ±0.04b	0.09 ±0.02a
1-Heptafluorobutyryloxydecane	-	19.771	nd	0.02±0.01	nd	nd
Tridecanol <n->	Musty	19.779	0.03 ±0.01	nd	nd	nd
Cycloheptasiloxane, tetradecamethyl-	-	20.038	0.18 ±0.04b	0.07 ±0.01a	0.13 ±0.03b	0.08 ±0.01a
2-methylhexacosane	-	20.092	nd	nd	0.09 ±0.01	nd
Eicosane	Waxy	20.100	0.07 ±0.01a	0.08 ±0.02a	nd	0.06 ±0.01a
Phenol, 2,4-bis(1,1-dimethylethyl-)	-	20.330	0.02 ±0.01	nd	nd	nd
Diethyl Phthalate	Odorless	20.995	0.21 ±0.08a	0.32 ±0.07a	0.24 ±0.06a	0.18 ±0.07a
Heptadecane, 2,6,10,15-tetramethyl-	-	21.390	nd	nd	0.01 ±0.001	nd
Cyclooctasiloxane, hexadecamethyl-	-	22.148	0.15 ±0.05a	0.10 ±0.03a	0.10 ±0.03a	0.07 ±0.03a
Tetradecanoic acid	Faint, waxy and fatty with a hint of pineapple and citrus peel	23.061	0.22 ±0.04b	0.11 ±0.02a	0.10 ±0.03a	0.07 ±0.03a
n-Nanodecanol-1	Fatty, waxy, floral, orange, sweet, clean, watery	23.361	0.02 ±0.01	nd	nd	nd
2-Propanol, 1-chloro-, phosphate (3:1)	-	23.531	0.07 ±0.01a	0.09 ±0.02a	0.07 ±0.01a	0.06 ±0.01a
Cyclononasiloxane, octadecamethyl-	-	23.605	0.17 ±0.04b	0.13 ±0.02b	0.08 ±0.02a	0.07 ±0.01a
Benzoic acid, hexyl ester	Fresh, balsam, sappy, clean, woody	23.816	0.04 ±0.01a	0.03 ±0.01a	0.03 ±0.01a	nd
Pentadecanoic acid	Waxy	23.875	0.02 ±0.01	nd	nd	nd
Morpholine, 4-octadecyl-	-	24.129	nd	0.05±0.02a	nd	0.02 ±0.01a
Hexadecanoic acid, methyl ester	Oily, waxy, fatty, orris	24.349	0.06 ±0.02a	0.02±0.01a	nd	nd
Cyclononasiloxane, octadecamethyl-	-	24.522	nd	nd	0.02 ±0.01	nd
Hexadecanoic acid <n->	Low heavy waxy, with a creamy, candle waxy nuance	24.574	0.59 ±0.11c	0.39 ±0.06b	0.27 ±0.07ab	0.20 ±0.06a
Cyclononasiloxane, octadecamethyl-	-	24.671	0.12 ±0.03b	0.03 ±0.01a	0.10 ±0.02b	0.07 ±0.02b
Cyclooctasiloxane, hexadecamethyl-	-	25.572	0.20 ±0.04b	0.09 ±0.02a	0.10 ±0.02a	0.06 ±0.02a
Octadecanoic acid	Oily, waxy	25.795	0.08 ±0.02a	nd	0.06 ±0.02a	0.02 ±0.02a
Stearic acid	-	25.805	nd	0.04±0.01	nd	nd
Cyclopropaneoctanoic acid, 2-octyl-, methyl ester	-	28.131	0.16 ±0.02	nd	nd	nd

13-Docosenoic acid, methyl ester, (Z)-		28.141	nd	0.03±0.01	nd	nd
Cyclononasiloxane, octadeca-methyl-	-	28.466	0.09 ±0.02a	0.10 ±0.02a	0.29 ±0.07b	nd

nd – not determined; Data expressed as a mean value (n = 3) ± standard deviation. [a-c] – Means with different letters within a row are significantly different ($p \leq 0.05$).

Table S4. Pearson correlations between overall acceptability and emotions response of the dark chocolate samples with volatile compounds.

Volatile acids	Overall acceptability		Happy		Sad		Angry		Surprised		Scared		Disgusted		Contempt		Valence	
	r	*p*	*r*	*p*	*r*	*p*	*r*	*p*	*r*	*p*	*r*	*p*	*r*	*p*	*r*	*p*	*r*	*p*
Acetic acid	0.527	0.078	-0.031	0.923	0.482	0.112	-0.019	0.952	-0.396	0.203	-0.294	0.354	.688*	0.013	-0.329	0.296	-0.101	0.756
Propanoic acid, 2-methyl-	.629*	0.028	-0.34	0.279	0.434	0.158	-0.013	0.967	-.826**	0.001	-.718**	0.009	.645*	0.024	-.704*	0.011	-0.368	0.239
2,3-Butanediol, [R-(R*, R*)]	-0.567	0.055	-0.053	0.871	0.214	0.505	.650*	0.022	-0.444	0.148	-0.044	0.893	-0.114	0.723	0.037	0.908	0.412	0.183
2,3-Butanediol	0.219	0.494	-0.403	0.194	-.709**	0.01	0.14	0.664	.825**	0.001	.750**	0.005	-0.544	0.067	0.301	0.341	-.616*	0.033
Acetic acid, ethoxyhydroxy- ethyl ester	.653*	0.021	-0.308	0.33	0.433	0.159	-0.081	0.802	-.801**	0.002	-.732**	0.007	.661*	0.019	-.701*	0.011	-0.369	0.239
Lactate <ethyl>	.655*	0.021	-0.307	0.332	0.436	0.156	-0.081	0.803	-.801**	0.002	-.731**	0.007	.665*	0.018	-.701*	0.011	-0.368	0.24
2,3-Butanediol, [S-(R*, R*)]	-0.494	0.103	.917**	0	0.307	0.332	-.725**	0.008	0.073	0.823	-0.281	0.375	0.163	0.614	0.232	0.468	.856**	0
Threo-3-bromo-2-pentanol	-0.474	0.119	-0.293	0.356	-0.077	0.811	.888**	0	-0.069	0.832	0.399	0.199	-0.397	0.202	0.27	0.396	0.133	0.679
Butanoic acid, 3-methyl-	-.813**	0.001	.749**	0.005	0.226	0.481	-0.206	0.521	0.058	0.858	-0.028	0.931	-0.108	0.739	0.388	0.212	.942**	0
Pentanoic acid, 3-methyl-	0.568	0.054	-0.34	0.28	0.334	0.289	-0.087	0.789	-.782**	0.003	-.738**	0.006	0.527	0.079	-.691*	0.013	-0.385	0.216
Butanoic acid, 3-methyl-	-0.503	0.096	.905**	0	0.292	0.356	-.726**	0.008	0.07	0.828	-0.286	0.368	0.157	0.625	0.223	0.486	.851**	0
Butyric acid <2-methyl->	0.487	0.109	0.27	0.396	0.099	0.759	-.873**	0	0.031	0.925	-0.417	0.177	0.406	0.19	-0.267	0.402	-0.136	0.673
1-Butanol, 3-methyl-, acetate	0.299	0.345	-0.323	0.305	-.676*	0.016	-0.084	0.796	.778**	0.003	.579*	0.049	-0.415	0.18	0.183	0.569	-.619*	0.032
L-lactic acid	.639*	0.025	-0.279	0.381	0.446	0.146	-0.111	0.731	-.809**	0.001	-.754**	0.005	.673*	0.016	-.702*	0.011	-0.341	0.279

Pentanoic acid	-0.007	0.983	.605*	0.037	0.014	0.966	-.982**	0	0.225	0.481	-0.319	0.312	0.213	0.506	-0.051	0.876	0.278	0.382
2-Butenoic acid, 3-methyl-	-0.517	0.086	.880**	0	0.265	0.405	-.723**	0.008	0.066	0.838	-0.292	0.357	0.153	0.635	0.205	0.522	.839**	0.001
2-Heptanol	-0.499	0.099	.910**	0	0.299	0.346	-.726**	0.008	0.071	0.826	-0.284	0.371	0.164	0.61	0.227	0.478	.853**	0
2-Hexanol, 5-methyl-	0.154	0.632	-0.445	0.148	-.725**	0.008	0.258	0.417	.822**	0.001	.812**	0.001	-.608*	0.036	0.331	0.293	-.603*	0.038
Pyrazine, 2,3-dimethyl-	-0.314	0.321	-0.256	0.421	-.753**	0.005	0.358	0.253	.855**	0	.880**	0	-.785**	0.002	0.498	0.099	-0.243	0.447
1-Methoxy-2-propyl acetate	.725**	0.008	-0.494	0.103	0.289	0.363	0.06	0.854	-.704*	0.011	-.588*	0.044	0.548	0.065	-.682*	0.015	-0.558	0.059
Pentanoic acid, 4-methyl-	-0.192	0.551	0.121	0.707	-0.199	0.536	0.224	0.483	.679*	0.015	.783**	0.003	-0.571	0.053	.768**	0.004	0.115	0.721
Benzaldehyde	.579*	0.049	-.884**	0	-0.473	0.121	0.483	0.111	0.142	0.661	0.324	0.305	-0.213	0.507	-0.223	0.485	-.967**	0
Hexanoic acid	-0.076	0.815	.755**	0.005	0.265	0.405	-.969**	0	0.026	0.936	-0.454	0.139	0.337	0.285	-0.027	0.933	0.5	0.098
Heptane, 2,2,4,6,6-pentamethyl-	-0.505	0.094	.901**	0	0.288	0.364	-.725**	0.008	0.07	0.83	-0.287	0.366	0.158	0.624	0.22	0.492	.850**	0
Cyclohexanol, 4-(1,1-dimethyl)-	-0.508	0.092	-0.318	0.314	-0.129	0.69	.880**	0	-0.076	0.813	0.385	0.217	-0.431	0.162	0.194	0.545	0.11	0.733
Cyclohexene, 3-(1-methylpropyl)-	.833**	0.001	-0.54	0.07	-0.21	0.512	-0.142	0.661	0.011	0.972	-0.096	0.766	0.192	0.55	-0.409	0.187	-.856**	0
Cyclotetrasiloxane, octamethyl-	0.091	0.78	0.546	0.066	.614*	0.034	-.716**	0.009	-.608*	0.036	-.870**	0	.688*	0.013	-0.388	0.212	0.449	0.144
Pyrazine, trimethyl-	-0.51	0.09	-0.333	0.291	-0.271	0.394	.655*	0.021	-0.085	0.793	0.252	0.43	-0.448	0.144	-0.103	0.75	0.009	0.979
(+)-4-Carene	-0.531	0.075	.837**	0.001	0.224	0.484	-.712**	0.009	0.059	0.854	-0.298	0.346	0.167	0.603	0.179	0.579	.815**	0.001
Octane, 2,3,3-trimethyl-	-0.51	0.09	-0.319	0.312	-0.132	0.684	.878**	0	-0.077	0.812	0.384	0.218	-0.432	0.161	0.189	0.555	0.109	0.736
Heptane, 2,5,5-trimethyl-	0.494	0.103	-0.35	0.265	0.257	0.42	-0.087	0.787	-.745**	0.005	-.718**	0.009	0.42	0.174	-.662*	0.019	-0.384	0.218

Decane, 2,3,4-trimethyl-	-0.023	0.943	-0.516	0.086	-.735**	0.006	0.485	0.11	.713**	0.009	.805**	0.002	-.662*	0.019	0.329	0.296	-0.531	0.076
2-Heptanol, acetate	-0.255	0.423	-0.431	0.162	-.634*	0.027	.603*	0.038	.633*	0.027	.865**	0	-.742**	0.006	0.384	0.217	-0.308	0.33
Pentane, 2,2-oxybis-	0.559	0.059	-0.342	0.276	0.323	0.305	-0.087	0.788	-.779**	0.003	-.736**	0.006	0.512	0.089	-.689*	0.013	-0.386	0.215
Pentadecane	.636*	0.026	-0.318	0.314	0.411	0.185	-0.083	0.798	-.801**	0.002	-.737**	0.006	.631*	0.028	-.703*	0.011	-0.375	0.23
Heptadecane	0.31	0.327	-0.318	0.314	-.666*	0.018	-0.083	0.798	.787**	0.002	.593*	0.042	-0.415	0.18	0.201	0.531	-.618*	0.032
Nonane, 5-(2-methylpropyl)-	-0.501	0.097	-0.313	0.322	-0.117	0.718	.884**	0	-0.075	0.817	0.389	0.211	-0.424	0.17	0.213	0.507	0.116	0.72
Ethanone, 1-(1H-pyrrol-2-yl)-	-0.464	0.128	0.537	0.072	-0.2	0.533	-0.555	0.061	0.477	0.117	0.162	0.615	-0.33	0.295	0.338	0.283	0.442	0.151
2-Pyrrolidinone	-0.524	0.08	-0.33	0.294	-0.159	0.621	.864**	0	-0.08	0.804	0.372	0.234	-0.447	0.145	0.144	0.655	0.095	0.77
Acetophenone	0.39	0.21	0.393	0.207	0.134	0.678	-.933**	0	0.051	0.874	-0.43	0.163	0.398	0.2	-0.199	0.534	-0.006	0.985
Heptanoic acid	.784**	0.003	-.782**	0.003	-0.242	0.448	0.314	0.32	-0.011	0.973	0.139	0.666	0.059	0.856	-0.335	0.287	-.943**	0
Pyrazine, 3-ethyl-3,5-dimethyl-	-0.506	0.093	-0.316	0.317	-0.124	0.701	.881**	0	-0.076	0.815	0.386	0.215	-0.428	0.165	0.201	0.531	0.112	0.728
Pyrazine, 3-ethyl-2,5-dimethyl-	0.025	0.939	0.262	0.411	-0.46	0.133	-0.536	0.073	.861**	0	0.458	0.134	-0.32	0.31	0.392	0.208	-0.083	0.798
H-Methyldodecanol	.596*	0.041	-0.333	0.29	0.364	0.245	-0.086	0.791	-.792**	0.002	-.741**	0.006	0.568	0.054	-.698*	0.012	-0.383	0.219
1-Heptanol, 2,4-diethyl	0.293	0.355	-0.326	0.302	-.680*	0.015	-0.084	0.794	.773**	0.003	0.571	0.053	-0.414	0.181	0.174	0.589	-.620*	0.032
2-Undecene, 2,5-dimethyl	-0.523	0.081	-0.33	0.295	-0.157	0.625	.865**	0	-0.08	0.805	0.373	0.233	-0.446	0.146	0.148	0.647	0.096	0.768
Pyrazine, tetramethyl-	.805**	0.002	-.586*	0.045	-0.3	0.343	-0.068	0.833	0.13	0.687	0.048	0.882	0.088	0.785	-0.328	0.297	-.895**	0
2-Nonanone	-0.499	0.099	-0.311	0.325	-0.113	0.726	.884**	0	-0.074	0.819	0.39	0.21	-0.421	0.173	0.218	0.496	0.118	0.716

Sulfurous acid, isobuthyl 2-pentyl ester	-0.499	0.099	.910**	0	0.298	0.346	-.726**	0.008	0.071	0.826	-0.284	0.371	0.165	0.608	0.227	0.479	.853**	0
1,6-Octadien-3-ol, 3,7-dimethyl-	.591*	0.043	-.914**	0	-0.295	0.352	.606*	0.037	-0.108	0.738	0.184	0.567	-0.086	0.791	-0.322	0.308	-.913**	0
Nonanal	-0.498	0.1	.912**	0	0.301	0.342	-.726**	0.008	0.072	0.825	-0.283	0.372	0.162	0.614	0.228	0.476	.854**	0
2-Isopryl-5-methylhes-2-enal	-0.48	0.114	-0.297	0.349	-0.086	0.79	.887**	0	-0.07	0.829	0.397	0.202	-0.403	0.194	0.257	0.419	0.129	0.688
Phenethyl alcohol	0.445	0.148	-0.104	0.747	-0.438	0.155	-0.444	0.148	.623*	0.03	0.285	0.37	-0.158	0.624	0.138	0.668	-0.497	0.1
1-Heptanol, 6-methyl-	-0.538	0.071	.802**	0.002	0.195	0.544	-.700*	0.011	0.055	0.866	-0.301	0.342	0.135	0.675	0.159	0.621	.793**	0.002
4H-Pyran-4-one, 2,3-dihydro-3,5-dihydroxy-6	.673*	0.017	-0.293	0.355	0.461	0.132	-0.078	0.81	-.796**	0.002	-.719**	0.008	.698*	0.012	-.694*	0.012	-0.357	0.254
Cyclopentasiloxane, decamethyl-	0.214	0.505	-0.212	0.508	-.661*	0.019	-0.112	0.729	.873**	0	.668*	0.018	-0.475	0.119	0.319	0.313	-0.505	0.094
Octanoic acid	0.34	0.279	0.243	0.447	-0.183	0.568	-.777**	0.003	0.423	0.171	-0.062	0.848	0.174	0.588	0.02	0.951	-0.16	0.619
3-Cyclohexen-1-ol, 4-methyl-1-(1-methylethyl)	-.678*	0.015	0.557	0.06	0.199	0.536	-0.098	0.763	0.02	0.951	-0.022	0.947	-0.005	0.988	0.436	0.156	.802**	0.002
Octanoic acid, ethyl ester	-0.47	0.123	0.128	0.693	-0.565	0.056	0.047	0.884	.849**	0	.721**	0.008	-.605*	0.037	.647*	0.023	0.136	0.673
Tetradecane <n>	.636*	0.026	-0.318	0.314	0.411	0.184	-0.083	0.798	-.801**	0.002	-.738**	0.006	.632*	0.027	-.703*	0.011	-0.375	0.23
Dodecane	-0.129	0.689	0.433	0.16	-0.382	0.22	-.662*	0.019	.775**	0.003	0.308	0.329	-0.249	0.436	0.357	0.254	0.12	0.709
Pentadecane <n>	-0.525	0.08	-0.331	0.293	-0.163	0.613	.861**	0	-0.081	0.803	0.369	0.238	-0.448	0.144	0.138	0.669	0.092	0.775
Estragole	0.386	0.215	0.354	0.259	0.123	0.703	-.914**	0	-0.013	0.969	-0.495	0.102	0.478	0.116	-0.335	0.287	-0.029	0.93
Sulfurous acid, decyl 2-ethylhexyl ester	.629*	0.029	-0.316	0.317	0.405	0.191	-0.082	0.799	-.794**	0.002	-.731**	0.007	.623*	0.03	-.696*	0.012	-0.372	0.234
Undecane, 2,6-dimethyl-	-0.538	0.071	.802**	0.002	0.195	0.544	-.700*	0.011	0.055	0.866	-0.301	0.342	0.135	0.675	0.159	0.621	.793**	0.002

Decane, 2,6,7-trimethyl-	-0.065	0.84	-0.492	0.104	-.641*	0.025	0.572	0.052	.634*	0.027	.779**	0.003	-.617*	0.033	0.393	0.206	-0.443	0.149
Thymoquinone	0.379	0.225	0.241	0.451	-0.002	0.995	-.831**	0.001	0.067	0.837	-0.392	0.208	0.442	0.15	-0.367	0.241	-0.123	0.703
Phenethyl isobutyrate	.606*	0.037	0.054	0.869	-0.07	0.83	-.719**	0.008	0.158	0.623	-0.253	0.427	0.375	0.23	-0.287	0.365	-0.374	0.231
Acetic acid, 2-phenylethyl ester	-0.508	0.092	-0.318	0.314	-0.127	0.693	.880**	0	-0.076	0.814	0.385	0.216	-0.43	0.163	0.196	0.542	0.111	0.732
Nonanoic acid	.636*	0.026	-0.088	0.786	-0.193	0.547	-.625*	0.03	0.216	0.5	-0.161	0.618	0.233	0.466	-0.297	0.349	-0.519	0.084
2H-Pyran-2-one, 5,6-dihydro-6-pentyl-	0.059	0.856	0.258	0.418	-0.413	0.182	-.579*	0.049	.767**	0.004	0.334	0.289	-0.238	0.457	0.373	0.232	-0.077	0.811
Hentriacontane <n->	-0.53	0.076	.836**	0.001	0.225	0.483	-.711**	0.01	0.06	0.854	-0.298	0.347	0.135	0.675	0.179	0.578	.814**	0.001
Dodecane, 4,6-dimethyl-	0.021	0.949	-.837**	0.001	-0.412	0.183	.931**	0	-0.027	0.932	0.405	0.192	-0.43	0.163	-0.087	0.787	-.583*	0.047
Phenol, 2-methyl-5-(1-methylethyl-)	-0.141	0.661	-0.329	0.296	-.633*	0.027	-0.115	0.723	0.272	0.392	0.092	0.775	-0.523	0.081	-0.067	0.835	-0.343	0.275
Nonane, 5-methyl-5-propyl-	-0.4	0.197	-0.24	0.452	0.005	0.987	.863**	0	-0.054	0.868	0.404	0.193	-0.327	0.3	0.377	0.227	0.163	0.612
Nonane, 3-methyl-5-propyl-	-.684*	0.014	.847**	0.001	0.293	0.355	-0.402	0.195	0.049	0.88	-0.127	0.694	-0.009	0.978	0.297	0.349	.918**	0
Pentadecane <n>	0.361	0.249	-0.276	0.384	-.587*	0.045	-0.075	0.818	.814**	0.001	.658*	0.02	-0.406	0.19	0.297	0.349	-.589*	0.044
Tridecane <n>	.701*	0.011	-0.216	0.5	0.534	0.074	-0.061	0.85	-.723**	0.008	-.624*	0.03	.788**	0.002	-.622*	0.031	-0.292	0.357
Thymol	-0.27	0.396	-0.166	0.605	-0.338	0.283	-0.199	0.536	-0.208	0.516	-0.436	0.157	-0.05	0.877	-0.317	0.316	-0.066	0.837
Octadecane, 1-(ethenyloxy-)	-0.43	0.163	-0.261	0.413	-0.025	0.938	.877**	0	-0.06	0.854	0.404	0.193	-0.354	0.258	0.339	0.28	0.153	0.635
Cyclohexasiloxane, dodecamethyl-	-.612*	0.035	.869**	0	0.149	0.643	-.606*	0.037	0.261	0.413	-0.07	0.828	0.007	0.983	0.382	0.22	.850**	0
Nonane, 3-methyl-5-propyl-	0.018	0.956	0.284	0.371	-0.397	0.201	-0.53	0.076	.834**	0.001	0.468	0.125	-0.297	0.348	0.439	0.154	-0.029	0.929

Eicosane	0.526	0.079	-0.347	0.269	0.289	0.362	-0.087	0.787	-.762**	0.004	-.728**	0.007	0.465	0.128	-.676*	0.016	-0.386	0.215
Dodecane, 4,6-dimethyl-	-0.526	0.079	-0.332	0.292	-0.164	0.611	.861**	0	-0.081	0.803	0.369	0.238	-0.449	0.143	0.136	0.673	0.092	0.776
1-Alanine, N-cyclopropyl-carbonyl-, methyl ester	0.032	0.921	0.047	0.885	-.599*	0.039	-0.407	0.189	.772**	0.003	0.387	0.214	-0.407	0.189	0.196	0.542	-0.259	0.415
3H-Pyrazol-3-one, 2,4-di-hydro-2,5-dimethyl	-0.4	0.197	-0.24	0.452	0.005	0.987	.863**	0	-0.054	0.868	0.404	0.193	-0.327	0.3	0.377	0.227	0.163	0.612
Sarcosine, N-cyclopropyl-carbonyl-, octyl ester	.693*	0.013	-0.244	0.444	0.509	0.091	-0.067	0.835	-.751**	0.005	-.660*	0.02	.757**	0.004	-.650*	0.022	-0.316	0.317
Decanoic acid <n->	-0.039	0.905	.705*	0.01	0.192	0.55	-.936**	0	0.15	0.642	-0.332	0.292	0.242	0.45	0.125	0.7	0.438	0.155
Propanoic acid, 2-methyl-, 3-hydroxy-2,4,4-tri	-0.089	0.783	.791**	0.002	0.354	0.259	-.935**	0	-0.069	0.831	-0.533	0.074	0.389	0.212	-0.024	0.942	0.56	0.058
Copaene	0.221	0.49	-0.347	0.269	-.718**	0.009	-0.087	0.787	.695*	0.012	0.466	0.127	-0.396	0.202	0.063	0.846	-.609*	0.036
Hexadecane, 1-iodo-	0.291	0.359	-0.327	0.3	-.683*	0.014	-0.085	0.794	.771**	0.003	0.567	0.055	-0.413	0.182	0.169	0.599	-.620*	0.032
Methylengenol	-0.049	0.881	0.172	0.592	-0.046	0.887	-0.524	0.08	-0.144	0.655	-0.503	0.096	0.181	0.574	-0.164	0.611	0.096	0.767
2,4,7,9-Tetramethyl-5-decyn-4,7-diol	0.151	0.638	0.483	0.112	0.089	0.784	-.587*	0.045	0.359	0.252	0.035	0.914	0.263	0.409	0.062	0.849	0.176	0.583
1-Heptafluorobutyryloxy-decane	0.485	0.11	-0.351	0.264	0.248	0.436	-0.087	0.787	-.740**	0.006	-.715**	0.009	0.408	0.188	-.659*	0.02	-0.384	0.218
Tridecanol <n->	-0.293	0.355	.915**	0	0.446	0.147	-.602*	0.038	0.089	0.783	-0.179	0.579	0.137	0.67	0.315	0.319	.771**	0.003
Cycloheptasiloxane, tetradecamethyl-	-0.5	0.098	.597*	0.04	-0.295	0.352	-.727**	0.007	0.493	0.104	0.005	0.988	-0.212	0.509	0.195	0.545	0.443	0.149
2-methylhexacosane	0.386	0.215	-0.24	0.452	-0.516	0.086	-0.067	0.837	.810**	0.001	.685*	0.014	-0.389	0.212	0.355	0.258	-0.552	0.063
Eicosane	-0.196	0.541	0.225	0.483	0.571	0.052	-0.042	0.896	-.794**	0.002	-.705*	0.01	0.44	0.152	-0.226	0.481	0.474	0.12
Phenol, 2,4-bis(1,1-di-methylethyl-)	-0.264	0.406	.887**	0	0.446	0.146	-0.571	0.052	0.088	0.786	-0.163	0.613	0.189	0.557	0.313	0.321	.739**	0.006

Diethyl Phthalate	.585*	0.046	-0.397	0.201	-0.013	0.967	-0.322	0.307	-0.502	0.096	-.662*	0.019	0.423	0.171	-.833**	0.001	-.581*	0.047
Heptadecane, 2,6,10,15-tetramethyl-	0.399	0.199	-0.083	0.797	-0.201	0.531	-0.03	0.926	.655*	0.021	.649*	0.022	-0.265	0.405	0.476	0.118	-0.341	0.278
Cyclooctasiloxane, hexadecamethyl-	-0.351	0.264	0.498	0.099	-0.035	0.913	-.777**	0.003	-0.041	0.899	-0.503	0.096	0.149	0.643	-0.15	0.642	0.428	0.165
Tetradecanoic acid	-0.443	0.149	.746**	0.005	0.148	0.647	-.806**	0.002	-0.014	0.966	-0.457	0.135	0.175	0.587	0.002	0.994	.673*	0.016
n-Nanodecanol-1	-0.522	0.082	0.528	0.078	0.012	0.971	-0.563	0.056	0.022	0.946	-0.284	0.371	0.079	0.807	0.035	0.913	.592*	0.042
2-Propanol, 1-chloro-, phosphate (3:1)	0.372	0.233	-0.299	0.346	0.11	0.735	-0.18	0.575	-0.53	0.076	-0.534	0.074	0.343	0.275	-.648*	0.023	-0.34	0.279
Cyclononasiloxane, octadecamethyl-	-0.147	0.648	.666*	0.018	0.442	0.15	-.785**	0.003	-0.369	0.238	-.733**	0.007	0.492	0.105	-0.167	0.604	.587*	0.045
Benzoic acid, hexyl ester	-.635*	0.027	0.055	0.865	0.09	0.781	0.256	0.421	-0.389	0.211	-0.208	0.517	-0.074	0.82	-0.001	0.997	0.461	0.132
Pentadecanoic acid	-0.522	0.082	0.528	0.078	0.012	0.971	-0.563	0.056	0.022	0.946	-0.284	0.371	0.079	0.807	0.035	0.913	.592*	0.042
Morpholine, 4-octadecyl-	0.522	0.082	-0.306	0.333	.581*	0.047	0.385	0.216	-.729**	0.007	-0.394	0.204	.637*	0.026	-0.325	0.302	-0.178	0.579
Hexadecanoic acid, methyl ester	-0.423	0.171	.637*	0.026	0.204	0.525	-.728**	0.007	-0.199	0.536	-0.559	0.059	0.238	0.457	-0.082	0.799	.636*	0.026
Cyclononasiloxane, octadecamethyl-	0.154	0.634	-0.35	0.265	-.717**	0.009	-0.086	0.79	.602*	0.038	0.362	0.248	-0.365	0.244	-0.028	0.93	-0.574	0.051
Hexadecanoic acid <n->	-0.244	0.444	.754**	0.005	0.337	0.284	-.879**	0	-0.201	0.531	-.622*	0.031	0.386	0.215	-0.148	0.647	.628*	0.029
Cyclononasiloxane, octadecamethyl-	-.714**	0.009	0.379	0.224	-0.544	0.067	-0.39	0.21	.616*	0.033	0.276	0.386	-0.537	0.072	0.275	0.387	0.357	0.255
Cyclooctasiloxane, hexadecamethyl-	-0.285	0.369	.824**	0.001	0.209	0.514	-.840**	0.001	0.192	0.549	-0.222	0.488	0.229	0.473	0.14	0.665	.652*	0.022
Octadecanoic acid	-0.411	0.184	.591*	0.043	-0.255	0.423	-.606*	0.037	.635*	0.027	0.204	0.524	-0.214	0.505	0.44	0.153	0.434	0.158
Stearic acid	.698*	0.012	-0.186	0.563	0.549	0.065	-0.054	0.867	-.685*	0.014	-.580*	0.048	.804**	0.002	-.586*	0.045	-0.264	0.407

Cyclopropaneoctanoic acid, 2-octyl-, methyl ester	-0.47	0.123	.937**	0	0.338	0.283	-.721**	0.008	0.077	0.812	-0.27	0.396	0.167	0.603	0.252	0.43	.860**	0.322
13-Docosenoic acid, methyl ester, (Z)-	0.42	0.174	-0.351	0.263	0.186	0.562	-0.086	0.789	-.697*	0.012	-.686*	0.014	0.321	0.31	-.624*	0.03	- 0.375	0.229
Cyclononasiloxane, octa-decamethyl-	0.463	0.13	-0.158	0.623	-0.474	0.119	-0.404	0.193	.617*	0.033	0.288	0.363	- 0.094	0.772	0.015	0.964	- 0.552	0.063

r – Pearson correlation; p – significance; correlation significant. when p≤0.05 (*) or p≤0.01 (**).
